# Phylogenetic structure and formation mechanism of shrub communities in arid and semiarid areas of the Mongolian Plateau

**DOI:** 10.1002/ece3.5787

**Published:** 2019-11-16

**Authors:** Ying Zheng, Lei Dong, Zhiyong Li, Jinghui Zhang, Zijing Li, Bailing Miao, Chengzhen Jia, Cunzhu Liang, Lixin Wang, Frank Yonghong Li

**Affiliations:** ^1^ Ministry of Education Key Laboratory of Ecology and Resource Use of the Mongolian Plateau & Inner Mongolia Key Laboratory of Grassland Ecology School of Ecology and Environment Inner Mongolia University Hohhot China; ^2^ State Key Laboratory of Vegetation and Environmental Change Institute of Botany Chinese Academy of Sciences Beijing China; ^3^ Meteorological Research Institute of Inner Mongolia Hohhot China; ^4^ Ecological and Agricultural Meteorological Center of Inner Mongolia Hohhot China

**Keywords:** cold limit, desert shrubs, paleoclimate, phylogenetic overdispersion, species coexistence

## Abstract

The mechanisms of species coexistence within a community have always been the focus in ecological research. Community phylogenetic structure reflects the relationship of historical processes, regional environments, and interactions between species, and studying it is imperative to understand the formation and maintenance mechanisms of community composition and biodiversity. We studied the phylogenetic structure of the shrub communities in arid and semiarid areas of the Mongolian Plateau. First, the phylogenetic signals of four plant traits (height, canopy, leaf length, and leaf width) of shrubs and subshrubs were measured to determine the phylogenetic conservation of these traits. Then, the net relatedness index (NRI) of shrub communities was calculated to characterize their phylogenetic structure. Finally, the relationship between the NRI and current climate and paleoclimate (since the Last Glacial Maximum, LGM) factors was analyzed to understand the formation and maintenance mechanisms of these plant communities. We found that desert shrub communities showed a trend toward phylogenetic overdispersion; that is, limiting similarity was predominant in arid and semiarid areas of the Mongolian Plateau despite the phylogenetic structure and formation mechanisms differing across habitats. The typical desert and sandy shrub communities showed a significant phylogenetic overdispersion, while the steppified desert shrub communities showed a weak phylogenetic clustering. It was found that mean winter temperature (i.e., in the driest quarter) was the major factor limiting steppified desert shrub phylogeny distribution. Both cold and drought (despite having opposite consequences) differentiated the typical desert to steppified desert shrub communities. The increase in temperature since the LGM is conducive to the invasion of shrub plants into steppe grassland, and this process may be intensified by global warming.

## Graphical abstract

Limiting similarity rather than habitat filtering is the main mechanism of community composition in temperate arid and semiarid areas desert. Low temperature is the main factor limiting shrub phylogeny distribution.

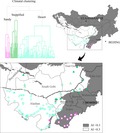

## 1 INTRODUCTION

The formation and maintenance mechanisms of community biodiversity have been an interesting theme in ecological research for several decades (Roughgarden, 1983; Tilman, 2004; Yang et al., 2011). With the rapid development of genetic information, gene sequencing, and computational power, evolutionary history combined with regional processes is used to understand the mechanism of species coexistence in biological communities (Cavender‐Bares, Kozak, Fine, & Kembel, 2009).

At present, two major mechanisms are recognized for species coexistence in communities: a deterministic process based on niche theory, and a stochastic process based on neutral theory. Niche theory emphasizes the role of niche differentiation and habitat filtering (Webb, 2000) and generally holds that species, which are phylogenetically closer share similar niches (Burns & Strauss, 2011). By calculating the phylogenetic distance of species within a community and comparing with the regional species pool null model, the phylogenetic community structure is determined (Webb, 2000). If the phylogenetic relationship between species within a community is closer than that predicted by the null model (i.e., phylogenetic clustering), habitat filtering is more important, since it has served to cluster species with similar niches. On the contrary, if more species with distant phylogenetic relationships appear than expected (i.e., phylogenetic overdispersion), limiting similarity (such as competitive exclusion and negative density dependent) is thought to be predominant (Webb, Ackerly, Mcpeek, & Donoghue, 2002). Neutral theory suggests that equal niches exist among different species and species composition depends on the constraints of stochastic processes and spatial distances (Bell, 2000).

Since Webb (2000) first applied the phylogenetic community structure to explain community construction, this concept has been widely used in the study of tropical rainforest and temperate forests (e.g., Döbert, Webber, Sugau, Dickinson, & Didham, 2017; Kraft, Valencia, & Ackerly, 2008; Letcher, 2010). However, the phylogenetic community structure of arid and semiarid shrub communities, especially temperate desert communities, is poorly understood to date. Environmental aridity could either serve as an environmental filter to cluster closely related species, or aggravate niche differentiation in order to use different resources (Cavender‐Bares et al., 2009). For example, Qian, Jin, and Ricklefs (2017) found that native communities in California were more phylogenetically clustered in dry areas, while Valiente‐Banuet and Verdú (2007) found that significant phylogenetic overdispersion of the communities in semiarid regions of Mexico was chiefly due to facilitation between distant species. Soliveres, Torices, and Maestre (2012) investigated 11 *Stipa* grassland communities in semiarid regions of Spain and suggested that most were phylogenetically random under the combination of habitat filtering and competitive exclusion, but that interaction between species (i.e., competitive exclusion) had a greater impact on community construction. Nevertheless, either facilitation or competition may result in phylogenetic overdispersion (Cavender‐Bares et al., 2009). Therefore, the role of habitat filtering in community construction (i.e., phylogenetic clustering) is not necessarily related to environmental stress. Under the premise of niche conservation, phylogenetic overdispersion could result from either competitive exclusion between close relatives or facilitation between distant species (Valiente‐Banuet & Verdú, 2007; Webb et al., 2002). Other researches indicated that low temperature was a strong habitat filter, especially for wood species (Qian, 2018; Qian, Hao, & Zhang, 2014). Yan, Yang, and Tang (2013) demonstrated an overall phylogenetic clustering on the Qinghai‐Tibetan Plateau and proved that both cold and dry climates were responsible for the formation of this lineage pattern. Thus, phylogenetic community structure in arid and semiarid regions may have different performance and formation mechanisms.

The western Mongolian Plateau is located in the interior of Eurasia beyond the reach of the warm and humid air currents of the Pacific and Indian oceans, and covered largely by sand and desert (Zhang, Hu, Zhuang, Qi, & Ma, 2009). With its ancient geological and floristic history, the region is rich in endemic and ancient remnant species, particularly in the Alashan‐Ordos region, which is regarded as one of the eight biodiversity centers of China (Wang, 2005; Zhu, Ma, Liu, & Zhao, 1999). Given that the study of phylogenetic structure combines regional environment, interspecific relationships, and geological history processes (Fine & Kembel, 2011), the western Mongolian Plateau constitutes an ideal location for phylogenetic study because of its ancient flora and harsh modern environment. In the present study, we took plant communities in the western Mongolian plateau as a model, for exploring species coexistence mechanisms and how phylogenetic structure responds to climate change in temperate arid and semiarid areas. We hypothesized that low temperature was more restrictive than drought for construction of shrub communities in temperate arid and semiarid areas, and that shrub communities would become phylogenetically more overdispersed under climate warming. This research will allow better understanding of the underlying mechanisms of community assembly and provide insight on biodiversity and ecosystem stability in arid and semiarid areas under global warming.

## 2 MATERIALS AND METHODS

### 2.1 Study area

**Figure 1:**
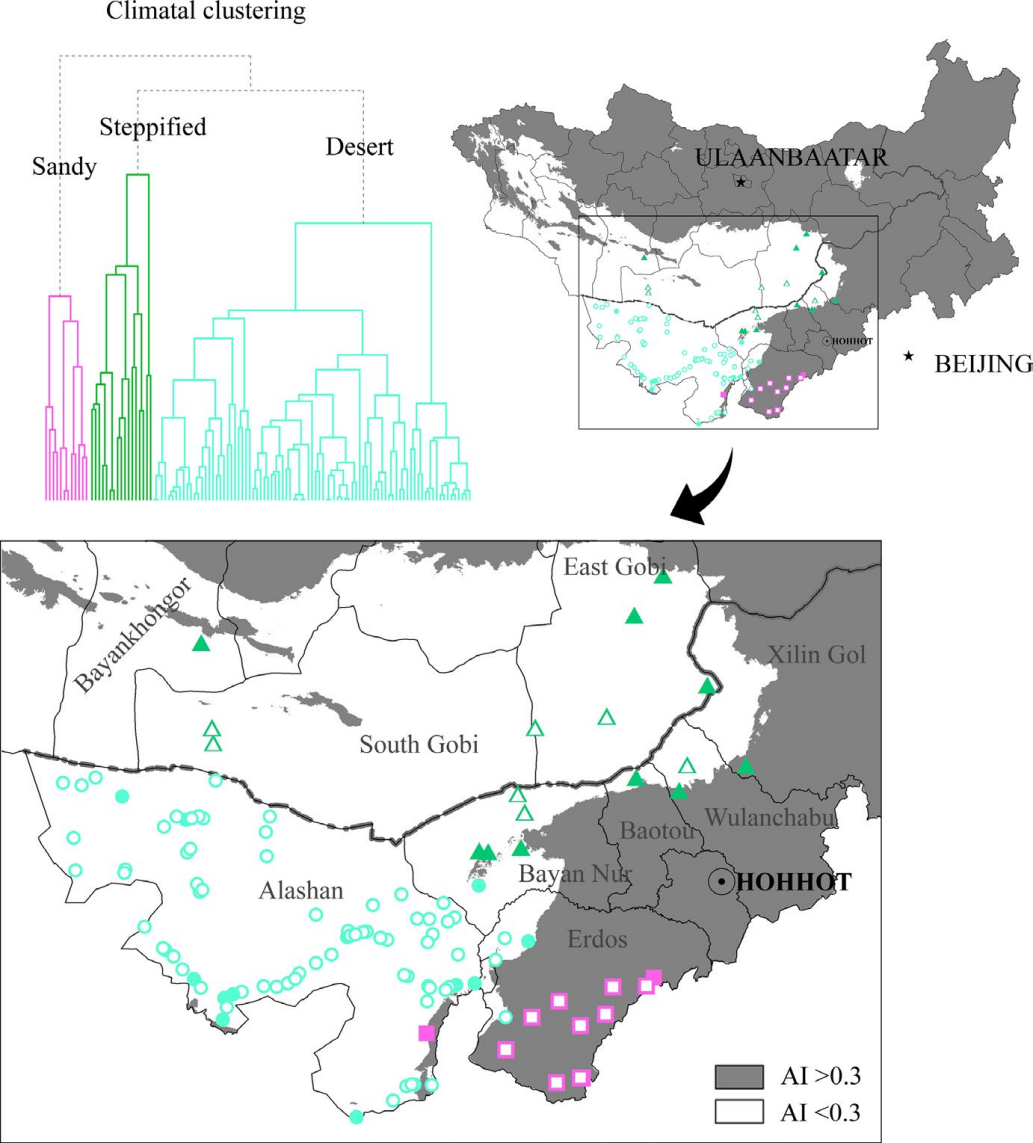
Distribution of surveyed communities, showing phylogenetically clustered (solid symbols) and overdispersed (outline symbols) shrub communities in arid and semiarid regions on the Mongolian Plateau (squares, triangles, and circles represent sand shrub, steppified shrub, and desert shrub, respectively)

The Mongolian Plateau, located in the hinterland of Asia, includes the whole territory of Mongolia, parts of southern Russia and most of China's Inner Mongolia Autonomous Region (Figure 1). The terrain is characterized by broad high plains and marginal mountains. Most of the plateau is at an altitude of between 1,000 and 1,500 m above sea level (John et al., 2018). It has a typical temperate continental climate, with precipitation falling mainly in summer, and is cold and dry in winter (Fang, Bai, & Wu, 2015). The eastern part of the Mongolian Plateau is covered dominantly by temperate steppes, the central and western parts by vast deserts (Liu et al., 2013). We focused our study on xeromorphic shrub and sandy shrub communities in arid and semiarid regions of the Mongolian Plateau. The average annual temperature and average annual precipitation (1979–2013) in this region range from −0.8 to 10.3°C and 37 to 426 mm, respectively. The plant communities in the region include desert steppe, steppified desert, and typical desert, successively distributed along the climatic gradient of increasing aridity (Wu, Zhang, Li, & Liang, 2015). The dominant shrubs are *Salsola laricifolia*, *Reaumuria soongarica*, and *Caragana stenophylla* in the steppified desert; *Nitraria sphaerocarpa*, *Potaninia mongolica*, and *Zygophyllum xanthoxylon*, in the typical desert; and *Artemisia ordosica* in the relatively humid Ordos Plateau. Additionally, many ancient endemic and endangered species are found in the western part of the plateau, the Erdos‐East Alashan desert area, such as *Tetraena mongolica*, *Helianthemum songaricum*, *Ammopiptanthus mongolicus*, *Amygdalus mongolica*, and *Gymnocarpos przewalskii* (Zhu et al., 1999).

### 2.2 Field survey

This study centered on Erdos‐Alashan and extended eastward to the desert steppe of the Wulanchabu Plateau and the western part of Xilin Gol in China's Inner Mongolia Autonomous Region, and East Gobi, South Gobi, and Bayankhongor in southern Mongolia. We surveyed 116 vegetation plots in total (Figure 1). Typical plant communities of local habitats with little human interference were selected. At each plot, we recorded all shrub and subshrub species within a 10 m × 10 m square; the height and canopy (length and width) of each shrub and subshrub were also measured, and latitude and longitude were recorded by GPS.

### 2.3 Determination and analysis of traits

Plant height, canopy area, leaf width, and leaf length were used to test trait conservation. The plant height and crown were measured with a tape measure. The canopy was approximated as an ellipse, and canopy area was thus calculated as canopy length × width × *π*/4. Height and canopy for each species in our study were taken as the average height and canopy area of the individuals measured (no fewer than 10 for most species). Leaf length and width data were derived from the median provided by the Flora Reipublicae Popularis Sinicae (FRPS, http://frps.iplant.cn/). For example, if the FRPS described the leaf width of *N. sphaerocarpa* as 2–4 mm, then the leaf width was set as 3 mm. Phylogenetic signals were measured using Pagel's *λ* model (Freckleton, Harvey, & Pagel, 2002; Pagel, 1999). The coefficient *λ* defines the weighting of phylogeny on traits. Under a completely Brownian motion model of evolution, phylogeny alone defines the expected covariance matrix of traits. However, as the influence of factors unrelated to phylogenetic history increases, the weighting of phylogeny will be reduced. *λ* is generally between 0 and 1, with values closer to 1 indicating that a trait is conserved (Münkemüller et al., 2012).

### 2.4 Phylogenetic analysis of plant communities

A list of all species recorded in our survey, and their general and family information based on the APG III (Angiosperm Phylogeny Group, 2009), was entered into the Phylomatic online plant database (https://phylodiversity.net/phylomatic/, storedtree = “Zanne et al. (2014)” and clean = “true”) to obtain a phylogenetic tree with differentiation time (Webb & Donoghue, 2005). It is worth noting that while gymnosperms such as *Ephedra kaschgarica* occupy an important position in some communities in the study area, the proportion of gymnosperms is quite small on a regional scale. However, the presence of gymnosperms strongly biases results (Feng et al., 2015; Letcher, 2010), so gymnosperms were removed in our final calculation to avoid distortion of the results at the broader scale.

The net relatedness index (NRI) was used to define the phylogenetic community structure (Webb et al., 2002). This index assumes that all species in the study area constitute a local species pool. First, the mean phylogenetic distance (MPD_observed_) of all species pairs in the sample is calculated. Then, without changing the number of species and the number of individuals in each species, the species in the sample are repeated randomly from the species pool 999 times to obtain the distribution of MPD of the random null model (i.e., MPD_rand_). Finally, the MPD_rand_ is normalized by stand diversion (SD). The NRI is calculated as follows:

$${\text{NRI}}_{\text{sample}}=-1\times \frac{{\text{MPD}}_{\text{observed}}-{\text{MPD}}_{\text{rand}}}{\text{SD}\left({\text{MPD}}_{\text{rand}}\right)}$$

In general, if NRI > 0, it means that the average phylogenetic distance between species in the sample is closer than the phylogenetic distance in the random null model; that is, the community is phylogenetically clustered. By contrast, if NRI < 0, the average phylogenetic distance between species in the sample is greater than in the random null model, and the community is phylogenetically overdispersed. If NRI ≈ 0, it indicates that the community is phylogenetically random (Swenson, Enquist, Jill, & Zimmerman, 2007).

### 2.5 Climate data

Climate data were taken from CHELSA (Climatologies at high resolution for the earth's land surface areas, http://chelsa-climate.org/), a global climate data set based on the time period 1979–2013. This contains 19 core bioclimatic data sets including annual mean temperature (AMT), mean annual precipitation (MAP), seasonal range of temperature variation, and precipitation in the driest quarter (defined as three consecutive months with least precipitation, generally between November and the following March; Table 1). More details and calculation methods of 19 climate factors were described by Karger et al. (2017). Potential evapotranspiration (PET) and aridity index (AI = MAP/PET) were downloaded from CGIAR‐CSI (http://www.cgiar-csi.org). PET and AI are averages calculated from meteorological data from 1970 to 2000 (Zomer, Trabucco, Bossio, & Verchot, 2008). A low AI value indicates a drier climate, with an AI of 0.3 usually defined as the boundary between arid and semiarid regions (Cardy, 1997). Paleoclimate data for the Last Glacial Maximum (LGM, about 0.021 Mya) were downloaded from Worldclim (http://www.worldclim.org/paleo-climate1), which is based on Community Climate System Model version 4 (CCSM 4). Reference to climate anomaly (current climate—LGM; Sandel et al., 2011) was used to represent climate change since the Last Glacial Maximum. The resolutions for all the bioclimatic data are 30 arcsec. Esri ArcGis 10.2 was used to extract the climate data for each sample by latitude and longitude.

**Table 1:** Twenty‐one climate factors and their implications

Climate factors	Description	Remarks
Bio 1	Annual mean temperature (AMT)	
Bio 2	Mean diurnal range (MDR)	
Bio 3	Isothermality (ISO)	Bio 2*100/Bio 7
Bio 4	Temperature seasonality (TS)	
Bio 5	Max Temperature of Warmest Month (TWM)	
Bio 6	Min temperature of coldest month (TCM)	
Bio 7	Temperature annual range (TAR)	Bio 5–Bio 6
Bio 8	Mean temperature of wettest quarter (MTW)	
Bio 9	Mean temperature of driest quarter (MTD)	
Bio 10	Mean temperature of warmest quarter (MTWA)	
Bio 11	Mean temperature of coldest quarter (TCQ)	
Bio 12	Annual precipitation (AP)	
Bio 13	Precipitation of wettest month (PWM)	
Bio 14	Precipitation of driest month (PDM)	
Bio 15	Precipitation seasonality (PS)	
Bio 16	Precipitation of wettest quarter (PWEQ)	
Bio 17	Precipitation of driest quarter (PDQ)	
Bio 18	Precipitation of warmest quarter (PWAQ)	
Bio 19	Precipitation of coldest quarter (PCQ)	
PET	Potential evapotranspiration (PET)	
AI	Aridity index (AI)	Bio 12/PET

### 2.6 Data analysis

All data analysis was performed in R version 3.5.1. The package “picante” was used to calculate abundance weighted (expressed as important value) NRI and Faith's phylogenetic diversity (Kembel et al., 2010). Principal component analysis (PCA) was performed on 21 climate data variables using the “princomp” function in R. The principal components with explanatory variables above 85% were selected, and the climatic distance between the samples was represented by the Euclidean distance on the selected principal component axis; the unweighted pair group method using arithmetic average (UPGMA) clustering (using the “hclust” function in R and set “method” = “average”) was then performed on the climate distance to divide climate habitats into groups. Spearman correlation coefficients between 21 climate factors and the NRI of each climate habitat group were calculated in order to identify the climatic factors with the greatest impact on each habitat group. The Wilcoxon signed‐rank test was used to test whether the NRIs were significantly different from zero. The Wilcoxon rank‐sum test was used to assess differences in NRI, PD, and species richness (SR) between habitat groups. Partial least squares regression (PLS) was used to analyze the power of climate factors in explaining NRI.

## 3 RESULTS

### 3.1 Phylogenetic signal

A significant phylogenetic signal (*p* < .05) was detected for leaf length and leaf width (with *λ* values of 0.505 and 0.758, respectively), but not for plant height (*λ* = 0.056, *p* = .754) or canopy (*λ* = 0.043, *p* = .794, Table 2).

**Table 2:** Phylogenetic signal test for shrub plant functional traits in western Mongolian Plateau

Traits	*λ*	*p*
Height	0.056	.754
Canopy	0.043	.794
Leaf length	0.505	.006
Leaf width	0.758	.014

### 3.2 Phylogenetic community structure

A total of 47 species of angiospermous shrubs and semishrubs were recorded in 116 samples in this study, belonging to 14 families and 29 genera according to the APG III system. The three families of Fabaceae, Amaranthaceae, and Asteraceae comprised 57.14% of the total species recorded (Table 3 and Table S1).

**Table 3:** Most commonly represented families and number of species recorded in shrub communities in western Mongolian Plateau

Family	Number of species	Percentage of total species	Representative genus
Fabaceae	11	23.40	*Caragana*
Amaranthaceae^a^	8	17.02	*Salsola*
Asteraceae	8	17.02	*Artemisia*
Others	11	42.55	*Reaumuria*, *Nitraria*
Total	47	100.00	—

a The Chenopodiaceae was incorporated into the Amaranthaceae in the APG III system.

**Figure 2:**
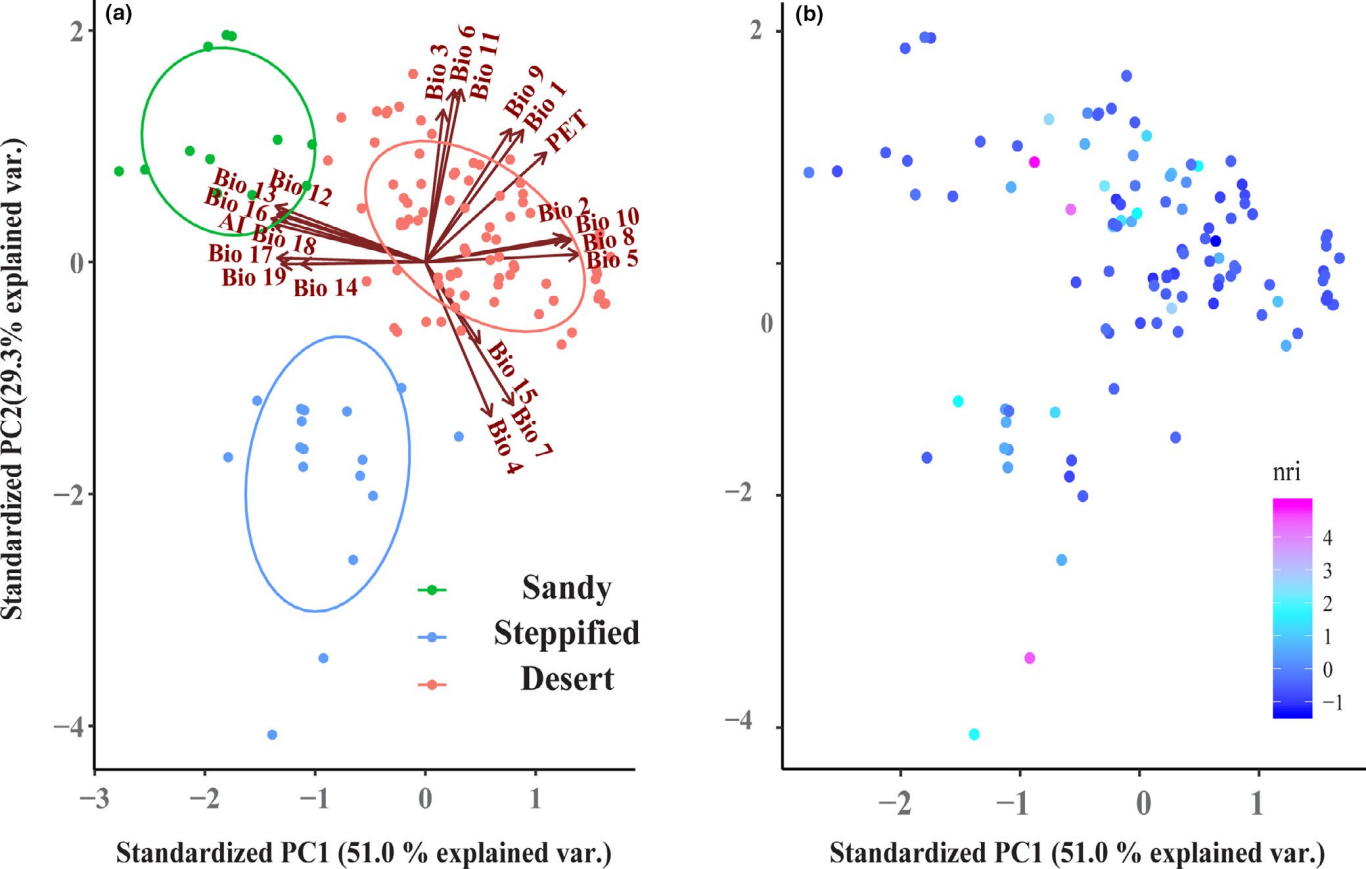
Distribution of relative climatic distance of points (a) and NRI (b) on the first two principal component axes. The first axis refers mainly to moisture conditions, the second axis to temperature. (Bio 1, annual mean temperature; Bio 2, mean diurnal range (mean of monthly max temperature–min temperature); Bio 3, Isothermality (Bio 2*100/Bio 7); Bio 4, Temperature Seasonality (standard deviation*100); Bio 5, Max Temperature of Warmest Month; Bio 6, Min Temperature of Coldest Month; Bio 7, Temperature Annual Range (Bio 5–Bio 6); Bio 8, Mean Temperature of Wettest Quarter; Bio 9, Mean Temperature of Driest Quarter; Bio 10, Mean Temperature of Warmest Quarter; Bio 11, Mean Temperature of Coldest Quarter; Bio 12, Annual Precipitation; Bio 13, Precipitation of Wettest Month; Bio 14, Precipitation of Driest Month; Bio 15, Precipitation Seasonality; Bio 16, Precipitation of Wettest Quarter; Bio 17, Precipitation of Driest Quarter; Bio 18, Precipitation of Warmest Quarter, Bio 19, Precipitation of Coldest Quarter)

The 21 climatic factors were decomposed by principal components, with the first three components representing 88.15% of the variation in all variables. The surveyed plots were clustered into three groups using UPGMA based on environmental distance: shrub communities in relatively warm and humid sandy habitats (“sandy shrub,” 12 plots), shrub communities in relatively dry and cold habitats in vegetation transition zones between desert and steppe, that is, desert steppe or steppified desert communities (“steppified shrub,” 17 plots), and typical desert shrub communities in extremely arid habitats (“desert shrub,” 87 plots; Figures 1, 2a, and Table S2).

**Figure 3:**
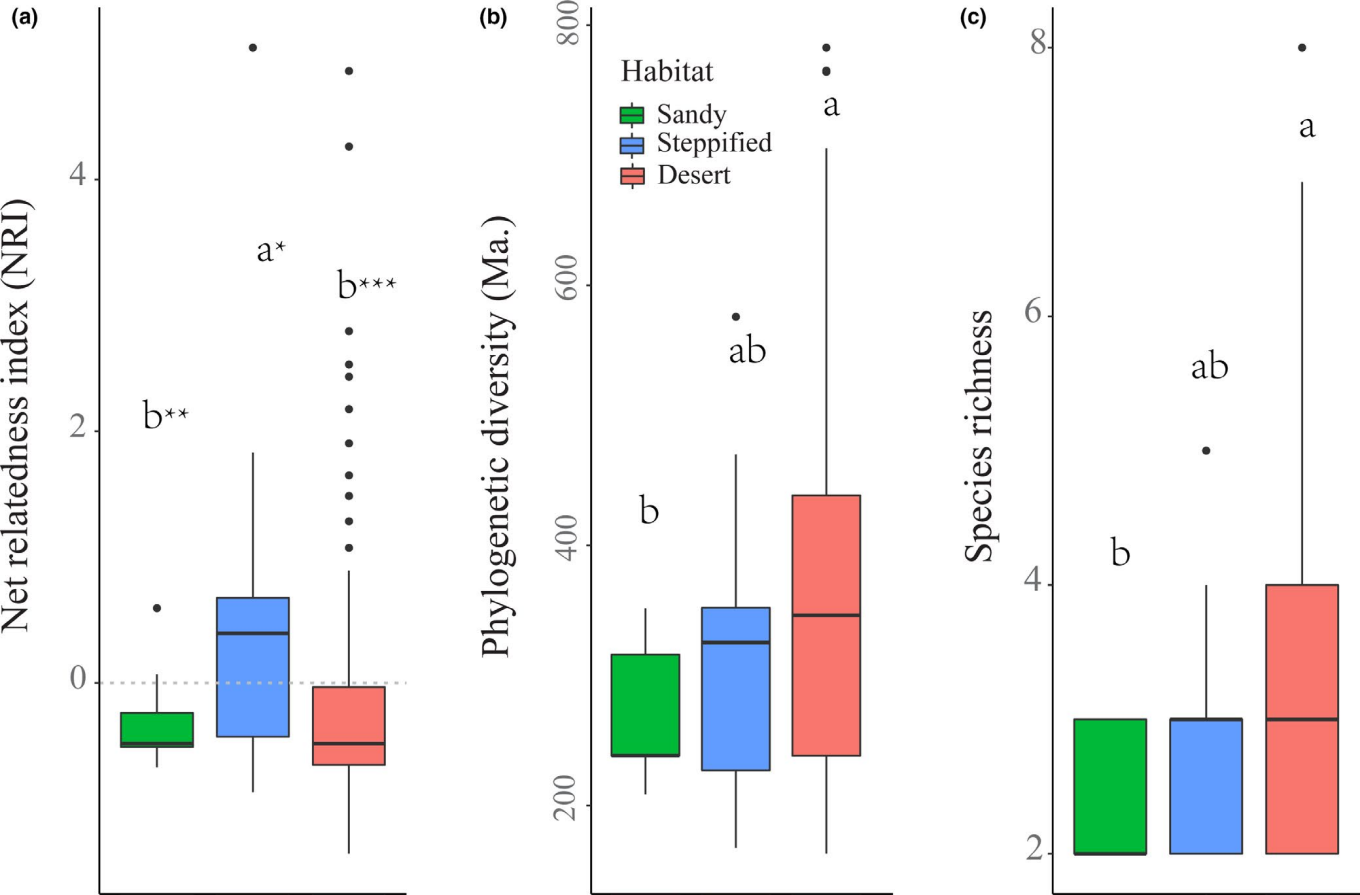
Net relatedness index (a), phylogenetic diversity (b) and species richness (c) of shrub communities in the three different habitats. Different letters indicate significant difference between communities in different habitats. Asterisks indicate NRIs significantly different from zero (*p < .1; **p < .05; ***p < .01)

The NRI of the studied shrub communities was significantly less than zero (*p* < .05), with phylogenetically clustered and overdispersed communities accounting for 29.66% and 70.34%, respectively. Sandy shrub and desert shrub communities showed significant phylogenetic overdispersion (*p* < .05), while steppified shrub communities showed some trend of phylogenetic overdispersion (*p* = .08). Phylogenetic diversity (PD) and species richness (SR) in desert shrub communities were significantly greater than in sandy shrub communities (*p* < .05), and somewhat higher than in steppified shrub communities (*p* = .557; Figure 3).

### 3.3 Relationship between current climate factors and phylogenetic community structure

**Figure 4:**
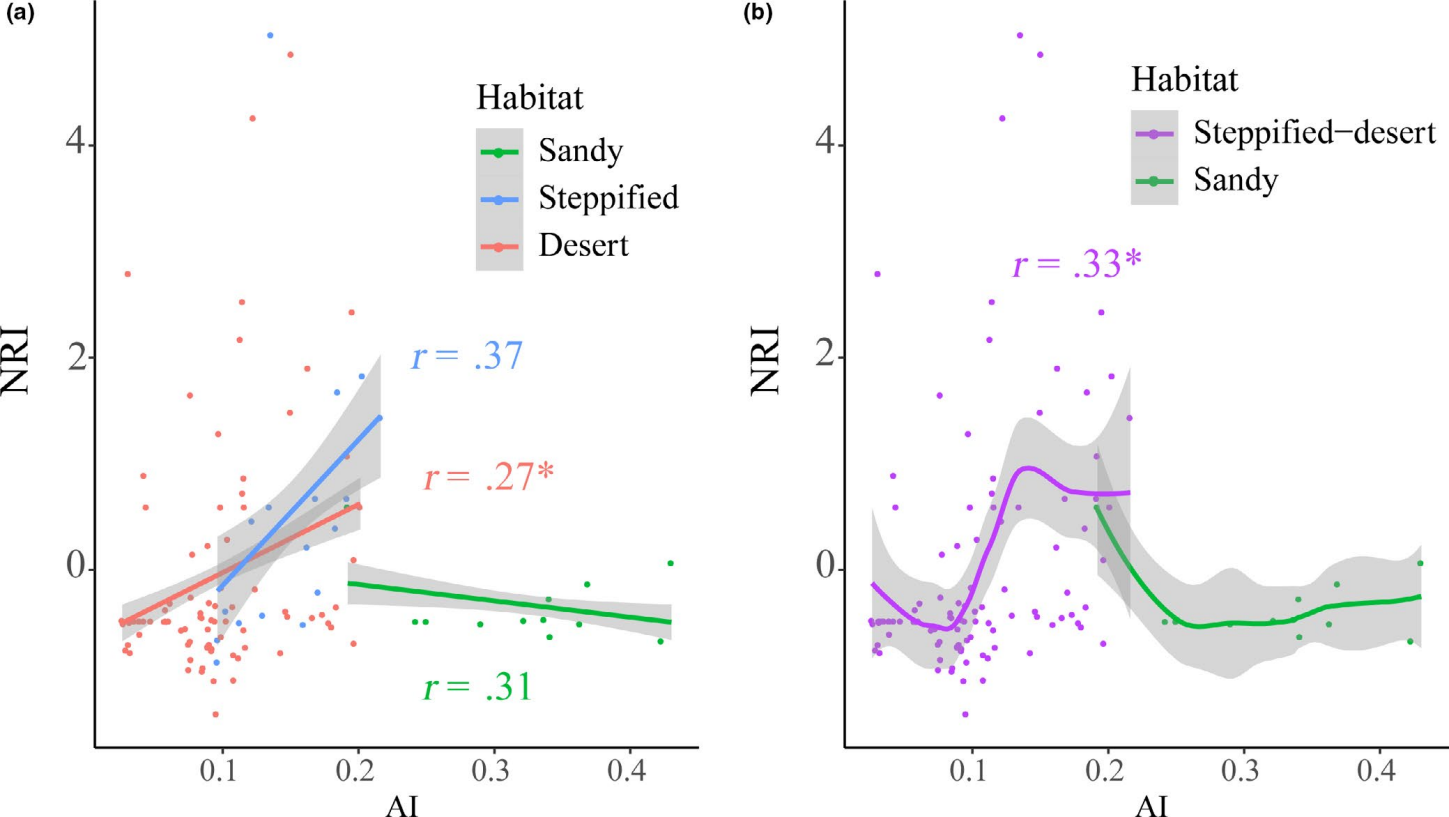
Changes in net relatedness index (NRI) of shrub communities in the three habitats along climate aridity (AI) gradient (*p < .01). (a) Linear regressions of sandy, steppified, and desert to AI; (b) smooth fitting of steppified‐desert and sandy to AI

**Figure 5:**
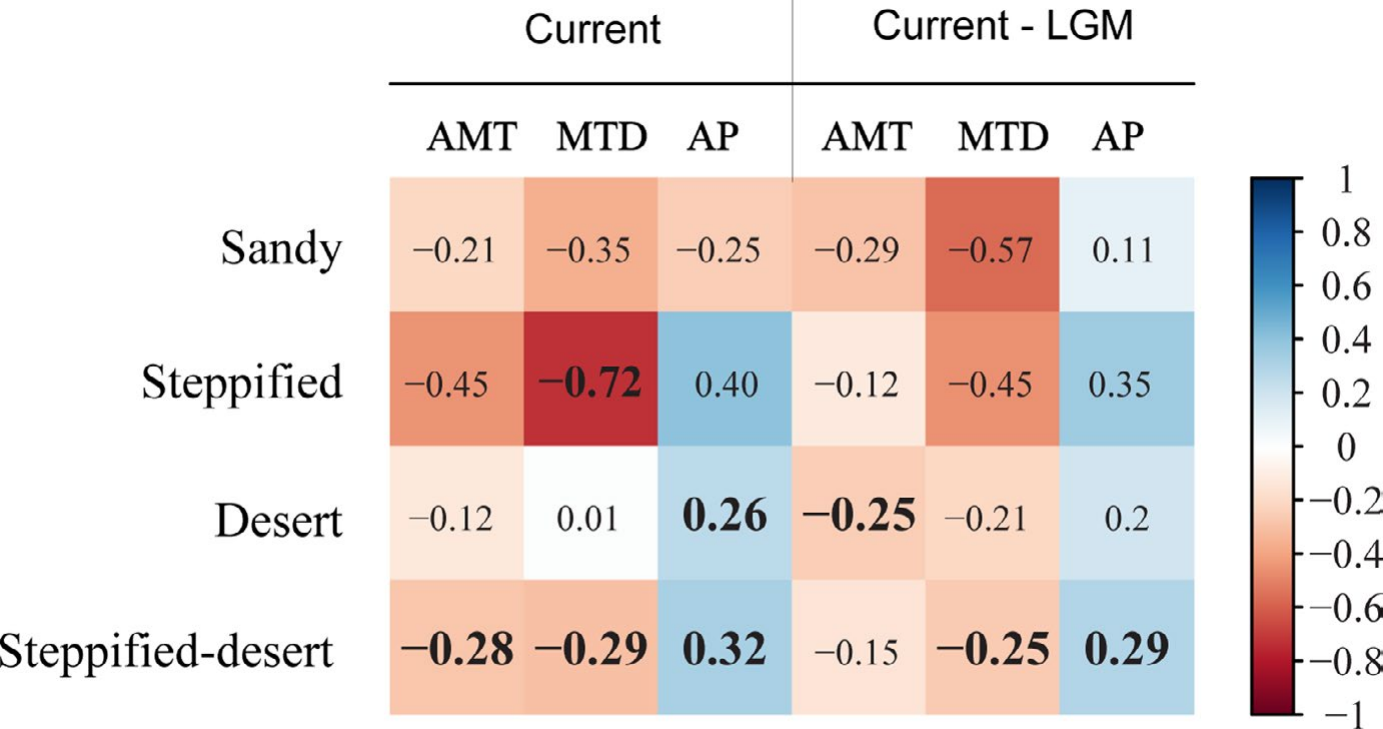
Spearman correlations between the NRI of shrub communities in different habitats and current climate as well as paleoclimate anomaly (AMT, Annual mean temperature; AP, Annual precipitation; MTD: Mean temperature of driest quarter). Significant values are in bold

The NRI of desert shrub and steppified shrub communities increased with increasing AI (*p* = .01 and 0.14 when analyzed, respectively [Figure 4a], or *p* < .01 when pooled together [Figure 4b]), whereas the NRI of sandy shrub communities showed a decreasing trend (not significant, *p* = .33; Figure 4a). In addition, Precipitation of Driest Month (Bio 14, PDM), Mean Temperature of Driest Quarter (Bio 9, MTD), and Precipitation of Driest Quarter (Bio 17, PDQ) were significantly correlated with the NRI of these shrub communities (*r* = .86 and *p* < .01 for sandy shrub, *r* = −.72 and *p* < .01 for steppified shrub, and *r* = .37 and *p* < .01 for desert shrub communities, respectively; Figure 5). Partial least squares regression (PLSR) analysis of the relations between community NRI and bioclimatic factors showed that climate factors had high capability in explaining NRI variation across sandy communities or steppified shrub communities, with the first three axes explaining 84.77% and 64.46% of NRI variation, respectively. Bioclimatic factors explained only 15.71% of NRI variation across desert shrub communities.

### 3.4 Relationship between paleoclimate anomaly and phylogenetic community structure

A comparison of current and paleoclimate data showed that AMT, MTD, and AP increased 6.37 ± 0.55°C, 5.38 ± 2.20°C, and 41.05 ± 21.28 mm, respectively, since the Last Glacial Maximum (LGM). The phylogenetic structures of sandy shrub and steppified shrub communities were less influenced by climate anomaly. The NRI of desert shrub communities was positively correlated with AMT anomaly from the LGM and that of steppified shrub communities was negatively related to MTD anomaly and positively related to AP anomaly since the LGM.

## 4 DISCUSSION

### 4.1 Significant phylogenetic conservation of shrub leaf traits

Niche conservation is a necessary assumption for applying phylogenetic analysis to community assembly (Kraft & Ackerly, 2010, Swenson, 2013). Leaf area (expressed by leaf length and width) strongly affects plant transpiration and water use (Cornelissen et al., 2003) for plant survival in arid and semiarid areas. Our results show that leaf traits of shrubs and semishrubs demonstrate a stronger phylogenetic signal than expected by the Brownian motion model, indicating that leaf width and length are phylogenetically conserved, and that closely related species have similar traits (Münkemüller et al., 2012). Plant height and canopy are important parameters for characterizing the ability of species to compete for light. Height and canopy are influenced by biotic and abiotic factors, that is, age and nutrient availability (Falster & Westoby, 2003). This may be one reason that height and canopy have no significant phylogenetic signal. Our results also suggest that height and canopy are more labile and may have better adaptability to changes in environment (e.g., climate warming). In contrast, leaf length and leaf width, due to their phylogenetic conservation, may restrict species survival.

Since leaf traits were not field measured, to avoid the autocorrelation of the data cited from the literature, we performed a phylogenetic signal test using height and crown data from the literature (not shown), which gave results identical to the measured data (i.e., no significant phylogenetic signal was detected). This indicates that the phylogenetic signals of leaf traits are more likely to correlate to phylogeny. In our study, we used only four traits to measure phylogenetic signal, which may provide incomplete information about phylogenetic conservation. Thus, more traits‐based research is needed to provide adequate evidence of phylogenetic conservation of traits in arid and semiarid shrub plants.

### 4.2 Phylogenetic structure and formation mechanisms of shrub communities

Our results show that the phylogenetic structures of shrub communities in the western Mongolian Plateau demonstrate a general trend toward phylogenetic overdispersion, though differences exist among the three groups corresponding to sandy, steppified desert, and typical desert habitats.

Desert shrub communities account for more than 3/4 of the sample communities in this study. The desert habitat is harsh with an extremely dry climate (annual precipitation generally < 200 mm); however, desert shrub communities show a significant phylogenetic overdispersion trend (Figure 3a), indicating the coexistence of distantly related species in these communities. Species competitive exclusion, facilitation between distantly related species, and other density‐dependent species interactions may contribute to the coexistence of distantly related species (Webb, Gilbert, & Donoghue, 2006), although it is difficult to isolate which process is responsible (Cavender‐Bares et al., 2009). Competition for below‐ground resources (groundwater especially) is key for plant survival when resources are limited (Wilson, 1988). Therefore, fierce competition for limited resources will inevitably make it difficult for species with similar ecological niches to coexist, and only species with differentiated niches can be retained in the community (Webb et al., 2002). As a result, communities were expected to be phylogenetically overdispersed. At the same time, climate factors explain only a small amount of the variation in NRI, further indicating that the phylogenetic structure of shrub communities in arid areas is less affected by (at least climate‐driven) abiotic environmental filtering (Dong et al., 2019). These results are supported by Qian et al. (2017) who found that compared to trees, shrubs and herbs showed less sensitivity to climate, and attributed this to their smaller size allowing them to take advantage of microhabitat protection.

As an example, *Reaumuria songarica* + *N. sphaerocarpa* is a widely distributed typical desert community. *Reaumuria songarica* is a shallow‐rooted plant with more lateral roots and most roots distributed in the range of 20–60 cm underground (Wu, Zhou, Zheng, Li, & Tang, 2014). In contrast, *N. sphaerocarpa* has deeper root distribution, enabling it to use groundwater below 80cm underground (Cui, Ma, Sun, Sun, & Duan, 2015). Coexisting species with differential water niches are expected, under the premise of a conservative niche, to be phylogenetically overdispersed (Webb et al., 2002). Our results were in contrast to those of Yan et al. (2013) on the Qinghai‐Tibetan Plateau, which showed an overall phylogenetic clustering. The possible reason for the discrepancy in the phylogenetic structures of shrub communities between these two plateaus may be difference in temperature. The elevation is much higher and the mean average temperature lower on the Qinghai‐Tibetan Plateau (over 4,000 m and AMT < 0°C and above 4,000 m) than on the Mongolian Plateau (below 1,740 m and MAT 8.7°C; Zheng, 1996), and low temperature rather than dryness is a strong filter for survival of woody plants (Qian et al., 2014). We will discuss this further below.

Steppified shrub communities show a weak phylogenetic clustering (*p* = .08), indicating an effect of habitat filtering on community construction. The weak (nonsignificant) phylogenetic clustering is most likely related to the fact that climate‐based habitat clustering cannot fully reflect shrub community structure. For example, some communities in this steppified shrub community group (phylogenetically overdispersed) show obvious desert shrub characteristics. Partial least squares (PLS) regression of NRI against climatic factors shows that community lineage in the semiarid area is strongly influenced by the mean temperature of the driest quarter (MTD, i.e., temperature in winter). That is, under the dual stress of low temperature (mainly) and drought, phylogenetic community structure shifts from overdispersion to clustering. The average annual temperature and the lowest winter temperature in the region where steppified shrub communities are predominate are the lowest among the three habitats: The temperature in winter often reaches −20°C, which is a challenge for shrub overwintering. Qian et al. (2014) showed that the woody phylogenetic community structure in a mountain ecosystem in China was limited by low temperature, tending toward phylogenetic clustering as temperature decreased. Qian (2018) also found that temperature (especially cold temperature) was more strongly correlated with angiosperm woody species in tropical South America. In addition, herbaceous plants play an important role in steppified communities, so that competitive pressure from these (biological filtration) may be the reason for community phylogenetic clustering. In recent years, shrub encroachment into grassland has attracted the attention of researchers (e.g., Chen et al., 2015; Eldridge et al., 2011). Our study highlights the effects of low temperature on the phylogenetic structure of steppified shrub communities and indicates that, as global temperature rises, that is, as low temperature stress reduces, the process of regional shrub encroachment may be aggravated (Chen et al., 2015; Moleele, Ringrose, Matheson, & Vanderpost, 2002).

Sandy shrub communities are distributed in markedly different habitats from desert or steppified shrub communities, in regions with higher precipitation (Figure 2a) and sandy substrates conducive to preserving precipitation and groundwater. The significant phylogenetic overdispersion of this group of shrub communities indicates that limiting similarity is the main driver of community construction (Kraft, Cornwell, Webb, & Ackerly, 2007). Species composition in sandy shrub communities is relatively simple, consisting of *A. ordosica* + leguminous shrub (e.g., *Caragana* sp. or *Corethrodendron lignosum*), with *A. ordosica*, the predominant species (Zhang, 1994). Phylogenetic overdispersion of plant communities may be related to negative density dependence, which limits distribution of the dominant species (Webb et al., 2006).

We combined desert and steppified shrub community types to examine the changes in shrub lineage along the aridity index (AI) gradient, and found a significant positive correlation between NRI and AI (Figure 4b). This result indicates that communities with phylogenetic clustering are more common in relatively wetter habitats, a phenomenon clearly shown in vegetation transition zones with a climate AI around 0.3 (see Figure 1). While only shrub plants were studied, herbaceous plants (synusia) in the steppified desert communities are generally developed, even dominant in some areas (Eldridge et al., 2011; Liu et al., 2015). The phylogenetic clustering of shrub communities may result from strong competition with herbaceous plants in this transition zone (Clary, Save, Biel, & De Herralde, 2004). A study in shrub‐encroached grasslands in Inner Mongolia showed that shrub patch density is negatively associated with precipitation and temperature and native vegetation (especially tall native herbs) is more likely to resist shrub encroachment (Chen et al., 2015). While herbs can occupy a large number of suitable niches, alternative niches for shrubs are limited and only a few species can survive in steppified shrub communities (Fowler, 1986). This is also consistent with shrub species richness and phylogenetic diversity being higher in desert shrub communities than in steppified shrub communities (Figure 3b,c). That is, biotic filtering plays an important role in forming shrub communities (Goberna, García, & Verdú, 2015). As climate becomes relatively more humid (i.e., AI continues to rise), the desert plant community is replaced by steppe community, and the dominant role of shrubs is also shared with herbs, except in sandy conditions (Fang et al., 2015).

### 4.3 Effect of paleoclimate and geological history on phylogenetic structure

Regional historical processes strongly affect species assembly and community phylogeny (Cavender‐Bares et al., 2009). That is, current community phylogeny is affected not only by the modern environment, but also by paleoclimate and geological process (Kissling et al., 2012; Valiente‐Banuet, Rumebe, Verdú, & Callaway, 2006). Sandel et al. (2011) showed that high climate‐change velocity since the Last Glacial Maximum is responsible for the global absence of endemism (e.g., amphibians, mammals, and birds). Variation between the Last Glacial Maximum (LGM) and the current climate has also had a high impact on the phylogenetic diversity structure of Chinese woody community lineage (Feng et al., 2014). Kissling et al. (2012) suggested that high paleoclimatic amplitudes, especially climate change since the Quaternary, are more likely to lead to phylogenetic clustering. However, our results unexpectedly show that NRI has a negative correlation with the lowest temperature anomaly (current MTD—LGM MDT), meaning that great temperature variation is conducive to phylogenetic overdispersion. Donoghue (2008) suggested that, when climate changes, it is easier (compared to in situ evolution) for species to track suitable habitat and migrate into a new area, providing there is a suitable corridor. Since there is no clear geographical boundary between steppified shrub and desert shrub communities, plants can gradually change their distribution with climate change, without becoming extinct. Since the last glacial period, the Mongolian Plateau has shown a clear trend toward becoming warmer and wetter. As minimum temperature has increased, the cold restriction on shrubs have been gradually relieved and shrub species would more easily move to the steppe area; thus, the lineage is overdispersed. By contrast, the increase in precipitation has increased the competitiveness of herbs, which in turn causes the opposite trend, phylogenetic clustering.

The typical desert area in this study belongs to the ancient Tethys region (Zhu et al., 1999) and has experienced a relatively stable arid climate since the late Tertiary (Liu, 1995). A large number of ancient Tethys flora and endemic Tertiary plants have been preserved in the area (Zhu et al., 1999). The long and stable evolutionary history and the existence of large numbers of endemic species provide abundant alternative regional species pools, rich in phylogeny with different niches (Anacker & Harrison, 2012) for community assembly (Cavender‐Bares et al., 2009; Pärtel, 2002). Therefore, this region has high pedigree and species diversity (Figure 3b,c), which may also be a cause of phylogenetic overdispersion of the desert shrub community (Gerhold, Pärtel, Liira, Zobel, & Prinzing, 2008).

## 5 CONCLUSIONS

The leaf traits of shrubs and semishrubs in arid and semiarid regions of the Mongolian Plateau are evolutionarily conserved, and phylogenetically closely related species have similar leaf traits. However, more trait‐based research is needed to provide further evidence for phylogenetic conservation of shrub traits on the Mongolian Plateau. Desert shrub and sandy shrub community lineage are dominated by overdispersion, with niche limiting similarity the main mechanism of community composition; shrubs in desert‐steppe and steppe‐desert transition areas are affected by herbaceous plant competition and restricted by low temperature, showing a trend of phylogenetic clustering. NRI tends to increase as climate aridity decreases. Difference in paleoclimate has opposite effects on community lineage structure; a high lowest temperature anomaly increases phylogenetic overdispersion, whereas a high precipitation anomaly increases the trend toward phylogenetic clustering. Increase in temperature under global warming is expected to lead to invasion of shrub plants into steppe regions.

## CONFLICT OF INTEREST

None declared.

## AUTHOR CONTRIBUTIONS

Ying Zheng, Lei Dong, Zhiyong Li, and Cunzhu Liang conceived the ideas. All authors coordinated the community survey. Lei Dong and Ying Zheng analyzed the data. Ying Zheng, Lei Dong, Zhiyong Li, Jinghui Zhang, Zijing Li, Bailing Miao, Chengzhen Jia, Cunzhu Liang, Lixin Wang, and Frank Yonghong Li wrote the paper.

## Data Availability

Data are in the Dryad Digital Repository https://doi.org/10.5061/dryad.6m905qfvv.
