# Supplementary material for: Phylogenetic structure and formation mechanism of shrub communities in arid and semiarid areas of the Mongolian Plateau
Source: Ecol Evol. 2019 Nov 16;9(23):13320–31. doi: 10.1002/ece3.5787 (PMC6912910; doi:10.1002/ece3.5787)
Supplement: Supplementary file 1 [file ECE3-9-13320-s001.docx]

Supplementary Table 1 Species list which were recorded in present study

| **Family** | **Genus** | **Species** |
| --- | --- | --- |
| Amaranthaceae | *Salsola* | *Salsola passerina* |
| Amaranthaceae | *Salsola* | *Salsola laricifolia* |
| Amaranthaceae | *Sympegma* | *Sympegma regelii* |
| Amaranthaceae | *Kalidium* | *Kalidium foliatum* |
| Amaranthaceae | *Anabasis* | *Anabasis brevifolia* |
| Amaranthaceae | *Salsola* | *Salsola arbuscula* |
| Amaranthaceae | *Krascheninnikovia* | *Krascheninnikovia ceratoides* |
| Amaranthaceae | *Kalidium* | *Kalidium cuspidatum* |
| Apocynaceae | *Periploca* | *Periploca sepium* |
| Asteraceae | *Artemisia* | *Artemisia xanthochloa* |
| Asteraceae | *Artemisia* | *Artemisia xerophytica* |
| Asteraceae | *Asterothamnus* | *Asterothamnus centrali-asiaticus* |
| Asteraceae | *Ajania* | *Ajania fruticulosa* |
| Asteraceae | *Artemisia* | *Artemisia ordosica* |
| Asteraceae | *Brachanthemum* | *Brachanthemum gobicum* |
| Asteraceae | *Artemisia* | *Artemisia sphaerocephala* |
| Asteraceae | *Ajania* | *Ajania achilleoides* |
| Caryophyllaceae | *Gymnocarpos* | *Gymnocarpos przewalskii* |
| Cistaceae | *Helianthemum* | *Helianthemum songaricum* |
| Convolvulaceae | *Convolvulus* | *Convolvulus gortschakovii* |
| Convolvulaceae | *Convolvulus* | *Convolvulus tragacanthoides* |
| Fabaceae | *Caragana* | *Caragana brachypoda* |
| Fabaceae | *Caragana* | *Caragana microphylla* |
| Fabaceae | *Caragana* | *Caragana tibetica* |
| Fabaceae | *Caragana* | *Caragana intermedia* |
| Fabaceae | *Caragana* | *Caragana korshinskii* |
| Fabaceae | *Caragana* | *Caragana stenophylla* |
| Fabaceae | *Oxytropis* | *Oxytropis aciphylla* |
| Fabaceae | *Corethrodendron* | *Corethrodendron scoparium* |
| Fabaceae | *Alhagi* | *Alhagi sparsifolia* |
| Fabaceae | *Ammopiptanthus* | *Ammopiptanthus mongolicus* |
| Fabaceae | *Corethrodendron* | *Corethrodendron lignosum var. laeve* |
| Nitrariaceae | *Nitraria* | *Nitraria sphaerocarpa* |
| Nitrariaceae | *Nitraria* | *Nitraria sibirica* |
| Nitrariaceae | *Nitraria* | *Nitraria tangutorum* |
| Polygonaceae | *Calligonum* | *Calligonum mongolicum* |
| Polygonaceae | *Atraphaxis* | *Atraphaxis frutescens* |
| Rosaceae | *Potaninia* | *Potaninia mongolica* |
| Rosaceae | *Amygdalus* | *Amygdalus mongolica* |
| Rosaceae | *Amygdalus* | *Amygdalus pedunculata* |
| Salicaceae | *Salix* | *Salix cheilophila* |
| Solanaceae | *Lycium* | *Lycium ruthenicum* |
| Tamaricaceae | *Tamarix* | *Tamarix chinensis* |
| Tamaricaceae | *Reaumuria* | *Reaumuria trigyna* |
| Tamaricaceae | *Reaumuria* | *Reaumuria soongarica* |
| Zygophyllaceae | *Zygophyllum* | *Zygophyllum xanthoxylon* |
| Zygophyllaceae | *Tetraena* | *Tetraena mongolica* |

Supplementary Table 2 Key climate variables (Mean ± SD) in different habitats

| Habitat | AMT (℃) | MTD (℃) | AP (mm) |
| --- | --- | --- | --- |
| Sandy | 8.28±0.71 | -8.07±1.15 | 317.08±71.86 |
| Steppified | 5.41±1.31 | -11.91±2.71 | 139.12±33.81 |
| Desert | 8.75±0.95 | -6.42±1.24 | 100.47±44.22 |

Note: AMT: Annual Mean Temperature, MTD: Mean Temperature of Driest Quarter, ap: Annual Precipitation.
